# Abdominal Obesity in Women with Polycystic Ovary Syndrome and Its Relationship with Diet, Physical Activity and Insulin Resistance: A Pilot Study

**DOI:** 10.3390/nu15163652

**Published:** 2023-08-20

**Authors:** Justyna Jurczewska, Joanna Ostrowska, Magdalena Chełchowska, Mariusz Panczyk, Ewa Rudnicka, Marek Kucharski, Roman Smolarczyk, Dorota Szostak-Węgierek

**Affiliations:** 1 Department of Clinical Dietetics, Faculty of Health Sciences, Medical University of Warsaw, E Ciołka 27, 01-445 Warsaw, Poland; jjaniszewska@wum.edu.pl (J.J.); dorota.szostak-wegierek@wum.edu.pl (D.S.-W.); 2 Department of Screening Tests and Metabolic Diagnostics, Institute of Mother and Child, Kasprzaka 17a, 01-211 Warsaw, Poland; magdalena.chelchowska@imid.med.pl; 3 Department of Education and Research in Health Sciences, Faculty of Health Science, Medical University of Warsaw, 00-581 Warsaw, Poland; mariusz.panczyk@wum.edu.pl; 4 Department of Gynecological Endocrinology, Medical University of Warsaw, Karowa 2, 00-315 Warsaw, Poland; ewa.rudnicka@poczta.onet.pl (E.R.); marekkucharski@outlook.com (M.K.); rsmolarczyk@poczta.onet.pl (R.S.)

**Keywords:** polycystic ovary syndrome, abdominal obesity, visceral adipose tissue, subcutaneous adipose tissue, insulin resistance, HOMA-IR, HOMA-AD, L/A ratio, physical activity, diet

## Abstract

Abdominal obesity is a common feature of women with polycystic ovary syndrome (PCOS), and it is known to exacerbate insulin resistance (IR). Improper dietary and physical activity patterns are crucial environmental factors involved in the development of obesity, and they can significantly influence the central deposition of adipose tissue. Therefore, in this cross-sectional study, we aimed to evaluate the relationship between abdominal adiposity (measured by VAT (visceral adipose tissue), SAT (subcutaneous adipose tissue), VAT/SAT ratio (visceral to subcutaneous fat ratio), and WHR (waist-to-hip ratio)) and the prevalence and odds ratios of IR (measured by the homeostatic model assessment of insulin resistance (HOMA-IR), the homeostatic model assessment-adiponectin (HOMA-AD) and leptin to adiponectin ratio (L/A ratio)) in 56 PCOS women. Furthermore, we investigated the relationship between these abdominal obesity indices and diet and physical activity. An original food frequency questionnaire and Actigraph GT3X-BT were used to assess adherence to the diet recommended in IR and the level of physical activity, respectively. We observed a higher prevalence of IR among women with higher VAT, VAT/SAT, and WHR values compared to women with normal values of those abdominal obesity indices. Moreover, VAT/SAT seemed to be the best predictor of IR measured by HOMA-IR and HOMA-AD. However, VAT appeared to be the best and strongest predictor of IR measured by the L/A ratio. We also observed that higher adherence to the diet recommended in IR and higher levels of vigorous physical activity were associated with lower values of central fat accumulation indices and a greater chance of their normal values. Our findings indicate that central obesity increases the odds of IR and supports the beneficial role of diet and physical activity in the management of abdominal obesity in PCOS women.

## 1. Introduction

Polycystic ovary syndrome (PCOS) is a common and heterogeneous condition among women of reproductive age. PCOS is characterized by anovulation, hyperandrogenemia (clinical or biochemical), and/or polycystic ovary morphology. In addition to these characteristics, hyperinsulinemia, insulin resistance (IR), and obesity are other important features of this syndrome. Numerous factors promote obesity in PCOS, including small or large size for gestational age, maternal PCOS, intrauterine hyperandrogenism, or menarche, which occurs too early or too late [1]. It is estimated that obesity is present in up to 75% of women with this endocrinopathy and may exacerbate the clinical and metabolic features of the disease. Additionally, it is observed that the central distribution of adipose tissue is particularly common in this group of women. A meta-analysis of 35 studies revealed that PCOS women had an elevated prevalence of central obesity (RR (relative risk) 1.73 95% CI 1.31–2.30; *p* < 0.05) compared to women without PCOS [2]. Moreover, the central accumulation of adipose tissue was found to be characteristic of both normal weight, overweight and obese women with PCOS and was associated with the disease regardless of BMI [3,4,5].

IR, defined as decreased responsiveness or sensitivity to the metabolic actions of insulin, is one of the most essential features of PCOS and affects up to 80% of its cases [6]. The early diagnosis and management of IR in women with PCOS is one of the most important aspects of preventing metabolic and hormonal complications. HOMA-IR (the homeostatic model assessment of insulin resistance) is the most commonly used marker for IR diagnosis in clinical practice. However, it is very often insensitive and may cause the underdetection of IR in PCOS cases [6,7]. HOMA-AD (the homeostatic model assessment-adiponectin) and L/A ratio (leptin to adiponectin ratio) are promising markers in the evaluation of IR in PCOS women because they concentrate on adipokines which are involved in the regulation of tissue insulin sensitivity [8,9,10]. Notably, apart from energy storage function, the adipose tissue is also a very crucial and active endocrine organ that secretes a variety of adipokines influencing the regulation of metabolism related to the sensitivity of tissues to insulin and reproductive function [11]. There is substantial evidence confirming that women with PCOS, regardless of obesity, are characterized by significantly lower concentrations of adiponectin compared to healthy women [8,12,13,14,15,16,17]. Leptin and resistin are other adipokines whose concentrations are abnormal in PCOS women. According to numerous authors, women with PCOS were characterized by an increased concentration of those adipokines, which further exacerbated the obesity-related disorders of tissue sensitivity to insulin [9,12,14,16,17,18]. Interestingly, it is subject to research whether those abnormalities in secreted adipokines in PCOS women are the direct result of abdominal obesity or whether they are rather related to PCOS.

The inter-relationship between obesity, IR, and endocrine abnormalities in PCOS is of complex nature. Obesity is known to exacerbate carbohydrate metabolism disorders and is an intermediate link between PCOS and IR. According to Li et al. [7], overweight and obese women with PCOS were at higher odds for IR (OR = 4.11, 95% CI 2.38–7.11, *p* < 0.001; OR = 10.99, 95% CI 5.82–20.76, *p* < 0.001, respectively) compared to lean PCOS subjects. However, a systematic review and meta-analysis of 28 studies highlighted that the diagnosis of PCOS independently of obesity was associated with a 27% decrease in insulin sensitivity [19]. Abdominal obesity particularly promotes hyperinsulinemia and IR in PCOS women, independently of the total quantity of body fat. It is considered that visceral adipose tissue is more strongly related to metabolic and hormonal disturbances in PCOS women than the subcutaneous one [5,11]. Visceral adipose tissue is regarded to cause disturbances in insulin signaling in women with PCOS and increase lipolysis, which encourages IR [3]. Additionally, excess abdominal adipose tissue is associated with low-grade chronic inflammation, which is another important element in the pathogenesis of IR in this endocrinopathy [20,21,22].

Recent recommendations regarding the treatment of PCOS highlighted the great importance of lifestyle interventions in the management of this disease and for weight control practice [23,24]. It was well-proven that a 5% reduction in body weight was associated with metabolic and hormonal improvements in the course of PCOS [23]. Improper dietary and physical activity patterns are crucial environmental factors involved in the development of obesity and IR in PCOS women, and they may significantly influence body composition, including the central deposition of adipose tissue. In particular, a poor-quality diet and low physical activity constitute additional risk factors for elevated abdominal adiposity apart from those associated with the pathophysiology of the disease [21,25].

Given the role of central abdominal obesity in the pathogenesis of IR in PCOS, in the present study, we aimed to investigate the relationship between the type of accumulation of body fat tissue (measured by VAT (visceral adipose tissue), SAT (subcutaneous adipose tissue), VAT/SAT ratio (visceral to subcutaneous fat ratio) and WHR (waist-to-hip ratio)) and the prevalence and odds ratios of IR (measured by HOMA-IR, HOMA-AD, and L/A) among PCOS women. Moreover, we analyzed the association between abdominal obesity, diet, and physical activity to establish the potential role of lifestyle interventions in the prevention and treatment of abdominal obesity in patients with PCOS.

## 2. Materials and Methods

### 2.1. Participants

This study included 56 women aged 18–40 years diagnosed with polycystic ovary syndrome according to the Rotterdam diagnostic criteria [26]. The participants were admitted to the Department of Gynecological Endocrinology of the Medical University of Warsaw in the years 2021–2022. The exclusion criteria were as follows: diabetes, thyroid dysfunction, endometriosis, Cushing’s syndrome, androgen-releasing tumor, congenital adrenal hyperplasia, chronic hypertension, cardiovascular diseases, the use of lipid-lowering, hormonal or insulin-sensitizing drugs, pregnancy, lactation and contraindications to body composition analysis (diagnosed epilepsy, implanted pacemaker or defibrillator and metal endoprostheses). For further analysis, the women were divided into four groups according to the visceral and subcutaneous fat content, visceral-to-subcutaneous fat ratio, and waist-to-hip ratio values. All the women provided written informed consent to participate in the study. The study was approved by the Ethics Committee of the Medical University of Warsaw (consent no. KB/170/2019).

### 2.2. Anthropometric Measurements

Body weight and height measurements were performed using standard procedures [27]. Body mass index (BMI) was calculated as follows: weight/height^2^ [kg/m^2^]. The interpretation of the values of this ratio was based on the classification published by the World Health Organization (WHO) [28]. Waist and hip circumferences were measured according to the WHO recommendations using stretch-resistant tape. Waist circumference was measured at the midpoint between the lower margin of the least palpable rib and the top of the iliac crest, while hip circumference was measured around the widest part of the buttocks. The waist-to-hip ratio (WHR) was calculated by dividing the waist circumference by the hip circumference. Abdominal obesity was defined as WHR ≥ 0.85 [29].

### 2.3. Body Composition Analysis with Bioelectrical Impedance (BIA)

In order to assess whole body composition, Maltron BioScan 920-II multi-frequency bioelectrical impedance analyzer was used (Maltron International Ltd., Rayleigh, UK). Before taking the BIA measurement, the women were instructed with the following guidelines provided by the European Society of Parenteral and Enteral Nutrition (ESPEN): no alcohol or fluids containing caffeine for 24 h before the test, lack of physical activity for 12 h before the test, empty stomach and bladder before the test [30]. The subjects underwent measurements according to manufacturer’s instructions in the supine position with the limbs separated 30 degrees away from the body axis after resting for about 3 min. The electrodes were placed on the top middle part of the right hand and on the top middle part of the right foot. To limit the possible errors and to ensure adherence, the sites were first cleaned with isopropyl alcohol [31].

In order to determine abdominal obesity, the quantitative analysis of subcutaneous and visceral tissue was performed in a standing position, with the upper limbs away from the body. The electrodes were placed in accordance with the manufacturer’s guidelines [31]. Based on waist circumference and the impedance of visceral and abdominal tissue, the following parameters were determined using Maltron BioScan 920 v. 1.1.135 software: subcutaneous fat surface (SAT in cm^2^), visceral fat surface (VAT in cm^2^) and visceral to subcutaneous fat ratio (VAT/SAT ratio). The cut-off for abdominal obesity was >120 cm^2^ for VAT and >225 cm^2^ for SAT. At the same time, the cut-off for VAT/SAT was defined above 0.9 [31].

### 2.4. Biochemical Analysis

Venous blood samples were obtained in the morning (between 7 a.m. and 9 a.m.) from each participant after 12 h fasting during the follicular phase (between days 2–6 of the menstrual cycle). In order to obtain the serum, the samples were centrifuged at 2500× *g* for 10 min at 4 °C. The serum for adipokine analyses was stored no longer than 3 months at −80 °C until being processed, while serum glucose and insulin results were determined on the day of blood collection.

Serum glucose level was assayed via the enzymatic method with hexokinase (Integra 400 plus analyzer, Roche Diagnostics, Basel, Switzerland), while serum insulin concentrations were analyzed using two-step chemiluminescent microparticle immunoassay (CMIA; Alinity I analyzer; Abbott Diagnostics GmbH, Wiesbaden, Germany). The concentrations of serum adipokines (adiponectin, leptin, and resistin) were determined by commercial enzyme-linked immunosorbent assay (ELISA), according to the manufacturer’s instructions. Leptin was assessed using a kit from DRG Instruments GmbH (Marburg, Germany). The analytical sensitivity of the assay was 0.7 ng/mL; intra-assay and inter-assay coefficients of variation (CV) were 4.2–7.3% and 3.7–9.1%, respectively. Total adiponectin was determined using a kit manufactured by TECOmedical AG (Sissach, Switzerland) with a lower detection limit below 0.27 ng/mL, intra-assay CV: 3.14–3.67%, and inter-assay CV: 6.93–8.16%. Resistin was measured using a kit manufactured by Mediagnost (Reutlingen, Germany) with the analytical sensitivity of the assay 0.012 ng/mL, intra-assay CV: 4.49–4.97%, and inter-assay CV: 3.37–6.67%.

The following mathematical models were used to establish insulin resistance: HOMA-IR (calculated as [fasting insulin (µU/mL) × fasting glucose (mg/dL)]/405); HOMA-AD (calculated as [fasting plasma insulin (μU/mL) × fasting glucose (mmol/L)]/adiponectin (μg/mL)) and L/A (calculated as leptin (ng/mL)/adiponectin (μg/mL)). The defined cut-off points were ≥2.5 for HOMA-IR [32], ≥6.26 for HOMA-AD [33], and >2.2 for L/A ratio [34].

### 2.5. Nutritional Assessment and Physical Activity Measurement

As we have already fully reported in our previous study [8], adherence to the diet recommended for insulin resistance was assessed with the original 15 food items food frequency questionnaire (FFQ) administered by a qualified nutritionist during a face-to-face interview. The participants were asked to report their average frequency (none, daily, weekly, or monthly) and the portion size of consumption for each food item. By assigning points 0, 0.5, or 1 for each item, adherence score was calculated.

For physical activity measurement, the participants wore an Actigraph GT3X-BT activity monitor (Actigraph Corp, Pensacola, FL, USA) at a belt around their waist, positioned on their right hip, over seven consecutive days. Raw accelerometer data were downloaded using ActiLife software (version 6.13.0, ActiGraph Corp, Pensacola, FL, USA). The duration of physical activity of moderate (MPA) or vigorous intensity (VPA), separately or combined (MVPA), was measured based on count thresholds corresponding to moderate or vigorous intensity activity. Freedson’s cut-off levels [35] were used to differentiate between the three intensity levels of physical activity: moderate physical activity (MPA): 1952–5724 counts/min, vigorous physical activity (VPA): ≥5725 counts/min and moderate + vigorous physical activity (MVPA) ≥ 1952 counts/min. Time spent in activity of a defined intensity (MPA, VPA, or MVPA) was determined by summing minutes in a week where the count met the criterion for the respective intensity.

### 2.6. Statistical Analysis

Statistical analyses were conducted using STATISTICA^TM^ 13.3 software (TIBCO Software, Palo Alto, CA, USA). A *p*-value of less than 0.05 was considered statistically significant. The Anderson-Darling test was used to evaluate variable distributions, and descriptive statistics were calculated, including the means, standard deviations, medians, and interquartile ranges. The comparison of women divided into four groups according to VAT, SAT, VAT/SAT, and WHR were performed with the Mann–Whitney–Wilcoxon test. Contingency tables with Fisher’s exact test were used to assess the relationship between the frequency and odds of insulin resistance in different abdominal obesity indices.

Linear regression analysis was employed to evaluate the relationship between abdominal obesity and physical activity or diet scores. Parameters for linear regression were estimated using the least square estimation method. A standardized regression coefficient (β) with a 95% confidence interval (95% CI) was calculated for each independent variable included in the model. Logistic regression analysis was conducted to examine the odds of normal values for the examined parameters based on diet or physical activity scores. Parameters for logistic regression were estimated using the maximum likelihood method. An odds ratio (OR) with a 95% CI was calculated for each independent variable included in the model.

## 3. Results

### 3.1. Baseline Clinical Features in Women with PCOS Divided According to the VAT, SAT, VAT/SAT Ratio and WHR

PCOS patients in each group were of similar age and height. As expected, the anthropometric and body composition data showed several differences between each group. The PCOS group with increased VAT (>120 cm^2^), SAT (>225 cm^2^), VAT/SAT ratio (>0.9), and WHR (≥0.85) had a significantly higher body weight level (*p* < 0.001 for each group), BMI (*p* < 0.001 for each group), waist circumference (*p* < 0.001 for each group), hip circumference (*p* < 0.001 for each group), total fat mass (*p* < 0.001 for each group) and muscle mass (*p* < 0.01 for each group) compared to the women with the normal values of those parameters.

Biochemical blood parameters also showed differences between groups, except for fasting glucose levels, which were not significantly different. We observed significantly higher levels of fasting insulin in the serum of women with increased VAT, SAT, VAT/SAT, and WHR (*p* < 0.001, *p* = 0.007, *p* < 0.001, and *p* = 0.005, respectively) compared to women with normal values of abdominal obesity indices. Additionally, in those groups of PCOS women, we also found lower adiponectin and higher leptin and resistin serum levels compared to those with the normal values of those parameters. Women with abdominal obesity had higher HOMA-IR and HOMA-AD values in contrast to women without abdominal obesity. We observed a similar relationship in the case of the L/A ratio.

Comparing diet scores between those groups of PCOS women, we found that women with increased VAT, VAT/SAT, and WHR values were characterized by significantly lower levels of adherence to the diet recommended in IR (measured by diet score) (*p* = 0.002, *p* = 0.023 and *p* = 0.026, respectively) as well as significantly lower levels of vigorous physical activity (*p* = 0.005, *p* = 0.026 and *p* = 0.027, respectively). Interestingly, we did not observe such differences in the case of groups that differed in subcutaneous fat content. Moreover, we observed no differences in the amount of moderate physical activity and the MVPA index in any group. Detailed comparisons of baseline clinical features of women divided into four groups are shown in Table 1. 

### 3.2. Abdominal Obesity and Insulin Resistance

We observed a significantly higher prevalence of IR measured by HOMA-IR, HOMA-AD, and the L/A ratio in PCOS women with increased visceral fat content, VAT/SAT ratio, and WHR value compared to those with the normal values of those parameters. Interestingly, a significantly higher IR frequency (measured by HOMA-AD and the L/A ratio) was observed in women with normal subcutaneous fat content compared to women with SAT > 225 cm^2^. However, those differences for IR measured by HOMA-IR were not statistically significant. Additionally, we noted the highest frequency of IR in PCOS women with abdominal obesity evaluated by the L/A ratio compared to HOMA-IR and HOMA-AD. Detailed data on the frequency of IR in women with PCOS and abdominal obesity are presented in Table 2. Moreover, we found that women with HOMA-IR ≥ 2.5 had higher VAT, SAT, VAT/SAT, and WHR values (z = −3.589; *p* < 0.001, z = −3.393; *p* < 0.001, z = −3.714; *p* < 0.001 and z = −3.294; *p* = 0.001, respectively). We found a comparable relationship for HOMA-AD ≥ 6.26 (z = −5.116; *p* < 0.001, z = −4.564; *p* < 0.001, z = −5.002; *p* < 0.001 and z = −3.637; *p* < 0.001, respectively) and the L/A ratio >2.2 (z = −5.795; *p* < 0.001, z = −5.654; *p* < 0.001, z = −5.236; *p* < 0.001 and z = −4.844; *p* < 0.001, respectively).

We found that the VAT/SAT ratio was the best predictor of IR measured by HOMA-IR and HOMA-AD. We observed that VAT/SAT > 0.9 significantly increased the odds of developing IR. When measured with HOMA-IR, they increased 41.12 times (OR 41.12, 95% Cl 2.27–743.52; *p* < 0.001), and with HOMA-AD, they increased 241.36 times (OR 241.36, 95% Cl 12.67–459.97; *p* < 0.001). VAT > 120 cm^2^ appeared to be the best and strongest predictor of IR measured by the L/A ratio (OR 68.44, 95% Cl 12.57–372.76; *p* < 0.001), followed by WHR ≥ 0.85 (OR 24.00, 95% Cl 5.48–105.05; *p* < 0.001) and SAT > 225 cm^2^ (OR 14.11, 95% Cl 1.62–122.70; *p* = 0.007), while VAT/SAT was found to be the most likely to increase the odds of abnormal HOMA-AD results. Interestingly, increased subcutaneous fat content appeared not to increase the odds of HOMA-IR ≥ 2.5. Detailed odds ratios for IR in relation to abdominal obesity are presented in Table 3.

### 3.3. Effect of Diet and Physical Activity on Abdominal Obesity

Linear regression analysis showed a differential effect of the diet score on abdominal obesity measured by VAT, SAT, VAT/SAT, and WHR. We observed that higher adherence to the diet recommended in IR (a higher diet score) was associated with lower VAT content (t = −2.635; *p* = 0.011), SAT content (t = −2.905; *p* = 0.005), and WHR value (t = −2.631; *p* = 0.011). However, no statistically significant relationship was determined between the diet score and the VAT/SAT ratio. Additionally, we noted that higher vigorous physical activity was associated with lower VAT content (t = −2.277; *p* = 0.027), SAT content (t = −2.028; *p* = 0.048), VAT/SAT ratio (t = −2.280; *p* = 0.027) and WHR (t = −2.421; *p* = 0.019). Conversely, no relationship was observed between moderate physical activity and MVPA and any of the fat distribution indices. Adjustment for BMI did not affect the results.

In addition, logistic regression showed a differential effect of the diet score on the odds of normal VAT content and WHR value. We observed that higher adherence to the diet recommended in IR translated into 43% greater odds of normal VAT content (OR 1.427, 95% Cl 1.091–1.868; *p* = 0.009) and 33% greater odds of normal WHR value (OR 1.325, 95% 1.023–1.716; *p* = 0.033). Interestingly, the diet score did not have an effect on the odds of normal SAT content or the VAT/SAT ratio. Additionally, higher vigorous physical activity was associated with greater odds of normal VAT (OR 1.063, 95% CI 1.007–1.122; *p* = 0.028) and VAT/SAT (OR 1.057, 95% CI 1.006–1.110; *p* = 0.028) values. However, no such relationship was observed in the case of vigorous physical activity or SAT content and WHR or between MVPA and any of the abdominal obesity indices. Similarly to the previous analysis, adjustment for BMI did not change the outcomes. Additionally, multivariate analysis showed that the diet score was a factor independent of physical activity that increased the odds of normal VAT content (OR 1.430, 95% Cl 1.097–1.864; *p* = 0.008), VAT/SAT (OR 1.273, 95% Cl 1.003–1.615; *p* = 0.047) and WHR (OR 1.322, 95% Cl 1.025–1.704; *p* = 0.031) ratios.

## 4. Discussion

It is widely acknowledged that PCOS women are more susceptible to the central accumulation of body fat compared to healthy BMI-matched counterparts. Abdominal obesity, distributed in subcutaneous areas as well as in the visceral parts of the abdomen, is present in 50–60% of cases of PCOS women [2,3,36,37]. Similarly to other papers, our previous study confirmed that PCOS women were characterized by a higher content of VAT and SAT compared to age-matched healthy women [4,5,8,37,38,39,40]. Other studies related to PCOS and based on waist circumference and WHR calculation also revealed that PCOS women had a greater tendency to accumulate body fat, predominantly in the central location [22,40,41]. Those results were confirmed by a systematic review and a meta-analysis of 47 studies. Higher accumulations of visceral fat (SMD (standardized mean difference) 0.41, 95% CI 0.23–0.59, *p* < 0.001) and abdominal subcutaneous fat (SMD 0.31, 95% CI 0.20–0.41, *p* = 0.008) were observed in PCOS women compared to BMI-matched controls [42].

BMI is the most widely accepted obesity indicator. However, it is not devoid of limitations. One of them is the inability to assess body fat distribution. Therefore, waist circumference (WC) and WHR are commonly used in clinical practice as more accurate indicators of abdominal adiposity. Nevertheless, they may be unable to reflect the volume and functions of adipocytes completely and accurately [41,43,44]. Therefore, computed tomography or magnetic resonance imaging seems to be a better method for assessing abdominal obesity due to the fact that they provide accurate information about the type and quantity of abdominal fat deposits [42]. In our study, apart from WHR, we also used bioelectrical impedance assessment for a more precise determination of the distribution and quantity of abdominal fat deposits (visceral and subcutaneous abdominal compartments). Furthermore, to evaluate abdominal obesity, we also proposed the VAT/SAT ratio, a measure of body fat distribution between VAT and SAT compartments, which is believed to be one of the best indicators of obesity-related metabolic comorbidities in men and women [43,45,46,47].

According to numerous authors, PCOS women had higher fasting insulin and HOMA-IR compared to healthy non-PCOS women [7,8,13,16,39,48]. Our previous investigation did not confirm this observation. However, the present study revealed that PCOS women with increased VAT, SAT, VAT/SAT, and WHR had significantly higher levels of fasting insulin in the serum and HOMA-IR compared to women with the normal values of abdominal obesity indices [8]. We also observed that other indicators of IR (HOMA-AD and the L/A ratio) were also higher in women with PCOS and abdominal obesity. Additionally, we noted that women with IR had significantly higher abdominal fat accumulation in contrast to women without IR. Our present results regarding abdominal obesity and IR are in accordance with previous findings by Chen et al. [11] and Mu et al. [36]. Wang et al. [49] evaluated abdominal obesity with VAI (Visceral Adiposity Index). They also reported that higher insulin levels and HOMA-IR values were observed in PCOS women with increased VAI values. Moreover, women with IR had significantly higher VAI values than women with normal insulin sensitivity. In a study by Mu et al. [36], normal-weight PCOS women with central obesity were at an increased risk of IR compared to their normal-weight non-centrally obese counterparts (OR 3.83, 95% CI 2.23–6.58; *p* < 0.001). Some other authors also demonstrated that IR was a more prominent feature in overweight and obese PCOS women than in lean PCOS women; however, without distinguishing the types of body fat distribution [40,44,50,51]. Our study also revealed a higher prevalence of IR measured by all three indicators in women with abdominal obesity (evaluated by VAT, VAT/SAT, and WHR). Interestingly, no such relationship was observed in the case of subcutaneous adipose tissue. A lower prevalence of IR was observed in PCOS women with an increased SAT content.

Our previous results stayed in line with the results obtained by other authors and confirmed that all IR indices correlated positively with abdominal obesity indicators. HOMA-IR, HOMA-AD, and the L/A ratio showed the strongest correlation with VAT content (r = 0.6061, *p* < 0.001; r = 0.7185, *p* < 0.001 and r = 0.7305, *p* < 0.001, respectively) compared to SAT and the VAT/SAT ratio [7,8,44,48,52]. However, other studies emphasized that WHR was also a parameter related to IR in PCOS women, specifically those assessed with the use of HOMA-IR [44,49,53]. Our present results revealed that VAT/SAT seemed to be the best predictor of IR measured by HOMA-IR and HOMA-AD. Conversely, VAT appeared to be the best and strongest predictor of IR measured by the L/A ratio. Moreover, apart from HOMA-IR, an increased SAT content was also associated with higher odds for IR. Research by Jena et al. [5] revealed similar results, as SAT was also not associated with HOMA-IR values. In contrast to those results, Tulloch-Reid et al. [54] demonstrated that SAT correlated with IR more strongly than with VAT in women. However, this study did not concern PCOS women. Our data are consistent with the findings of the Framingham Heart Study conducted in 3223 men and women, where the VAT/SAT ratio was associated with a risk of IR to a larger extent than VAT alone [47]. Using a multivariate linear regression model, Ng et al. [38] observed that abdominal obesity measured by waist circumference was an independent predictor of IR in PCOS women. Several studies demonstrated that VAI was the best predictor of metabolic syndrome and IR in PCOS women [49,55,56], while others indicated that WHR was also a useful insulin sensitivity predictor [53]. In the Framingham Heart Study [57] concerning the association between VAT and SAT compartments and metabolic risk factors among men and women, it was pointed out that both types of adipose tissue were associated with increased odds of metabolic syndrome. However, the relationship was stronger for VAT than SAT, even after adjustment for potential confounders. Our study also showed a weaker but still influential association between the odds of IR (measured by HOMA-AD and the L/A ratio) and SAT accumulation in PCOS women. Despite the fact that SAT seems to be less associated with metabolic alterations in PCOS women than VAT, VAT/SAT, or WHR, it is still a metabolically active compartment of abdominal adipose tissue that should not be overlooked. Therefore, all types of abdominal fat distribution must be clearly analyzed when assessing the relationship between metabolic disorders in PCOS women.

There are numerous structural, functional, and prognostic differences between VAT and SAT. Due to the fact that VAT is more metabolically active and shows a greater lipolytic activity, it is more strongly associated with the pathogenesis of IR, T2DM (type 2 diabetes mellitus), and other metabolic disorders than SAT [37,38,46]. Currently, it is considered that SAT is also linked to metabolic complications in PCOS women. Therefore, it should be pointed out that different fat compartments contribute to IR risk to varying degrees [58]. Disparate results regarding the prevalence of IR and SAT accumulation obtained in our study may be partially explained by the fact that some authors reported that SAT might exert protective effects against cardiometabolic alterations [59,60]. Interestingly, a prospective cohort study by Porter et al. [60] conducted in men and women demonstrated that a possible protective effect of SAT on cardiometabolic risk factors existed only in those in the highest tertile of VAT accumulation. The protective role of SAT may be explained by the fact that this adipose tissue contains larger adipocytes and secretes more cardioprotective adipokines and smaller quantities of pro-inflammatory markers than the visceral one. Notably, VAT and SAT compartments differ in the secretion of metabolically active adipokines. SAT secretes more leptin and adiponectin, whereas VAT secretes more resistin, visfatin, interleukin-6, interleukin-8, and plasminogen activator inhibitor-1 than SAT does [10,60]. Our previous research stayed in line with other papers, which revealed that adiponectin levels were inversely correlated with BMI, total body fat, and the markers of abdominal obesity, whereas a positive correlation was observed for leptin and resistin levels [8,12,13,18,61]. We also observed that all three adipokines showed the strongest correlation with VAT [8].

Adiponectin is one of the most crucial adipokines which regulates insulin sensitivity, and its concentration is negatively correlated with HOMA-IR. Moreover, adiponectin presents anti-inflammatory, antineoplastic, and cardioprotective properties [12]. In a study by Cardoso et al. [62], adiponectin concentration decreased with an increase in body fat percentage in PCOS women. Moreover, obesity, especially the visceral one, is known to decrease the expression level of adiponectin receptors and reduce its postreceptor signaling [21]. Leptin and resistin are other adipokines involved in insulin sensitivity management. Hyperleptinemia and an increased level of resistin are associated with obesity and result in impaired glucose metabolism and IR [12]. It is well established that women with PCOS may experience dysregulation in the synthesis of adipokines in the adipose tissue [62]. We previously reported that PCOS women were characterized by significantly lower adiponectin levels compared to non-PCOS women, while leptin and resistin did not differ between those two groups [8]. In contrast to those results, the present study showed that leptin and resistin levels differed significantly when we stratified PCOS women based on abdominal adiposity indices. PCOS women with abdominal obesity were characterized by lower adiponectin concentrations but also higher leptin and resistin levels. Our results are consistent with those of other authors, where leptin and resistin levels were increased in obese PCOS women [12,16,63]. However, it is still under investigation whether such abnormalities are the result of obesity, IR, or hormonal disorders or if they constitute independent symptoms of PCOS. The results of our study may suggest that leptin and resistin levels are strongly correlated with abdominal obesity, and their secretion is upregulated in increased abdominal adiposity among PCOS women, whereas hypoadiponectinemia is associated with PCOS diagnosis independently of abdominal obesity. Therefore, more research is needed to determine a definite answer to this question in the future.

The importance of diet and physical activity in the management of obesity in PCOS is well documented, and lifestyle modifications are recommended as the first-line therapeutic element in weight management strategies in women with this endocrinopathy [23,24]. It is regarded that the Mediterranean diet (MD) and the Dietary Approaches to Stop Hypertension diet (DASH) are associated with a lower risk of abdominal obesity in men and women in the general population [64,65,66,67,68]. In particular, dietary fiber was found to be inversely correlated with abdominal adiposity, whereas simple carbohydrates, dietary trans, and saturated fats were characterized by a positive correlation [69]. The data regarding the relationship between individual products, as well as the MD and DASH diet and abdominal obesity in PCOS, are missing. However, Rodrigues et al. [25] reported that poor quality diet was related to overweight and obesity, also the abdominal one (measured by WC) in PCOS women. They also suggested that the quality, rather than the quantity, of consumed food was a more important factor affecting body composition. Our study supports those results. We observed that a dietary pattern (evaluated by the diet score), which was similar to the MD and DASH diet, rich in wholegrain cereal products with a low glycemic index, vegetables, fruits, legumes, nuts, natural yogurt, vegetable oils, and fatty sea fish, and poor in red meat, especially processed, and products that were a source of simple sugars, disaccharides, and trans fatty acids seemed to have a beneficial effect on abdominal obesity indices. Higher adherence to this dietary pattern (a higher diet score) was associated with lower VAT content, SAT content, and WHR value (no relationship with VAT/SAT). Moreover, a higher diet score was associated with greater odds of a normal VAT content and WHR value (SAT and VAT/SAT were not related). Regrettably, women with abdominal obesity were characterized by significantly lower compliance with this dietary pattern. A study by Ehsani et al. [70] revealed that a diet rich in fried vegetables, vegetable oils (except olive oil), salty snacks, legumes, eggs, fast foods, onion, and garlic, and poor in sweets, high/low-fat dairy products, cruciferous vegetables, simple sugars, and honey was positively related to VAI, another abdominal obesity indicator in PCOS women. Moreover, they noted that each one-degree increase in compliance with this diet was associated with two times greater odds of visceral tissue dysfunction (OR 2.77, 95% CI 1.15–6.66, *p* < 0.05). Interestingly, another study by Alissa et al. [71] showed that a very diverse diet (a high diet diversity index) in women with PCOS was associated with a higher risk of abdominal obesity (measured by WHR). The authors explained their observations by the fact that a large number of products in the diet could lead to the consumption of excessive amounts of energy during the day. In our research, the diet score included low glycemic index products, which seemed to have an inverse relationship with abdominal obesity. A study by Melekoglu et al. [72] partially confirmed this observation. In PCOS women, low glycemic load was inversely associated with the WHR value. Furthermore, low glycemic index was unrelated to abdominal adiposity index (such a relationship was observed in a group of healthy women). A study by Graff et al. [73] revealed that a high glycemic index was associated with higher WC in PCOS women. Conversely, a study by Goss et al. [74] revealed that a reduced-carbohydrate diet (41% of energy from carbohydrates) for eight weeks resulted in the reduction of SAT (−7.1%) and VAT compartments (−4.6%). Research by Pasquali et al. [75] demonstrated that a hypocaloric diet added to metformin treatment in PCOS women with abdominal obesity resulted in the reduction of WC and visceral depots (SAT and VAT/SAT ratio remained unchanged). Similar findings regarding the reduction of abdominal obesity were observed in a PCOS group in a study by Zhang et al. [76] (VAT) and Marzouk et al. [77] (WC). It is worth mentioning that the VAT compartment is believed to be more susceptible to reduction than SAT due to the greater metabolic and lipolytic activity. Our study supports the importance of a particular dietary pattern in the management of abdominal obesity in PCOS women. However, future studies should focus on the relationship between individual dietary factors, which can potentially modify the risk of abdominal adiposity in this group of women.

Data regarding the relationship between abdominal fat distribution and physical activity in PCOS women are scarce. Our study is one of the few that examines the relationship between physical activity and abdominal obesity in a group of women with PCOS. Several studies conducted in the general population indicated the beneficial influence of physical activity on the central accumulation of body fat [78,79]. A study by Ando et al. [79] demonstrated that a sedentary lifestyle was associated with VAT accumulation, whereas standing time was inversely related to VAT. In our research, we noted that higher vigorous physical activity levels were associated with lower VAT content, SAT content, VAT/SAT ratio, and WHR. Furthermore, higher vigorous physical activity was associated with greater odds of normal VAT and the VAT/SAT value (no relationship with SAT and WHR). Moreover, women with abdominal obesity were characterized by significantly lower vigorous physical activity than non-centrally obese women. However, MVPA was not related to abdominal obesity indices in PCOS women. Other authors supported our findings, but they evaluated abdominal obesity with WC measurements [80,81,82]. An interventional study by Hutchison et al. [39] demonstrated that 12 weeks of intensified aerobic exercise resulted in the reduction of visceral fat (−12.0 cm^2^, *p* = 0.03), while no significant change was observed in the control group. On the contrary, SAT was significantly decreased in control women, which was not observed in women with PCOS. Our data stay in line with the results of other studies and indicate that low physical activity is an important contributor to abdominal obesity among PCOS women. Bearing in mind our previous report, in which we demonstrated that physical activity was a crucial environmental factor involved in IR management in PCOS women, we may assume that the improvement of tissue insulin sensitivity in physically active women may be significantly modulated by abdominal obesity [8].

The strength of our study was the precise assessment of abdominal fat distribution using the Maltron BioScan 920-II multi-frequency bioelectrical impedance analyzer rather than only anthropometric surrogate measures of body fat. Moreover, another strength was the use of an accelerometer for the precise measurement of physical activity. However, the study findings should be interpreted in light of some limitations, with the small sample size being the first one. Secondly, the use of an original food frequency questionnaire based on frequency and portion sizes declared by the participants might lead to the underestimation or overestimation of dietary intake. The third limitation might be linked to the use of the surrogate measures of IR measurement instead of a hyperinsulinemic euglycemic clamp—the gold standard technique. Finally, our population was only Caucasian, which could significantly reduce the representativeness of the study.

## 5. Conclusions

In conclusion, our study indicates that PCOS women with abdominal obesity may have increased odds of IR (evaluated by HOMA-IR, HOMA-AD, and the L/A ratio) compared to non-centrally obese PCOS women. Moreover, an increased VAT/SAT ratio and VAT seem to be better predictors of IR among PCOS women than SAT or WHR. SAT is also an important abdominal compartment associated with the risk of IR. However, much more weakly linked to impaired insulin sensitivity. Taken together, the assessment of abdominal adiposity should be performed to better predict IR in PCOS, and all abdominal indices should be considered in analyzing the relationship between abdominal fat distribution and the risk of metabolic complications. Additionally, diet and physical activity are linked to reduced IR risk, probably, among others, by modulating the abdominal obesity status in PCOS women. The modification of diet and an increase in physical activity seem to be promising methods in the treatment of insulin resistance in PCOS, especially in abdominally obese PCOS patients. However, studies in a larger group of PCOS patients are particularly needed to establish the role of abdominal fat compartments in the management of IR in PCOS and its relationship with diet and physical activity.

## Figures and Tables

**Table 1:** Baseline clinical features in women with PCOS divided according to VAT, SAT, VAT/SAT ratio and WHR.

Parameters	VAT ≤120 cm *n* = 31	VAT >120 cm^2^ *n* = 25	*p*-Value *	SAT ≤225 cm^2^ *n* = 47	SAT >225 cm^2^ *n* = 9	*p*-Value *	VAT/SAT ≤0.9 *n* = 29	VAT/SAT >0.9 *n* = 27	*p*-Value *	WHR <0.85 *n* = 35	WHR ≥0.85 *n* = 21	*p*-Value *
AGE AND ANTHROPOMETRIC MEASUREMENTS												
Age (years)	25.65 ± 4.46	26.36 ± 3.65	NS	26.28 ± 4.26	24.33 ± 2.78	NS	25.66 ± 4.55	26.3 ± 3.61	NS	25.6 ± 3.85	26.57 ± 4.51	NS
	25	26		25	24		25	26		25	26	
	19–38	21–36		19–38	21–29		19–38	21–36		19–35	21–38	
Weight (kg)	60.01 ± 7.96	95.82 ± 15.41	<0.001	70.51 ± 17.86	104.64 ± 15.23	<0.001	60.39 ± 8.39	92.76 ± 18.32	<0.001	66.55 ± 17.66	91.73 ± 17.91	<0.001
	58	94		66.7	111		58.9	93		59	91	
	45–75	70–126.4		45–119	83.7–126.4		45–75	56–126.4		45–117	63–126.4	
Height (cm)	166.26 ± 4.27	167.24 ± 6.07	NS	166.83 ± 5.1	166 ± 5.48	NS	166.45 ± 4.35	166.96 ± 5.92	NS	166.91 ± 5.03	166.33 ± 5.38	NS
	165	165		165	165		166	165		166	165	
	158–174	158–180		158–180	159–174		158–174	158–180		158–178	159–180	
BMI kg/m^2^	21.65 ± 2.69	34.2 ± 5.03	<0.001	25.21 ± 5.88	37.92 ± 4.97	<0.001	21.73 ± 2.82	33.18 ± 6.05	<0.001	23.77 ± 5.91	33.06 ± 5.86	<0.001
	21.2	35.5		23.5	39.3		21.5	34.5		22.3	34.50	
	17.2–27.8	24.8–42.9		17.2–36.7	28.7–42.9		17.2–27.8	21–42.9		17.2–42.9	23.4–41.7	
WC (cm)	72.77 ± 7.96	104.28 ± 10.94	<0.001	81.94 ± 15.24	112.44 ± 9.96	<0.001	73.07 ± 8.58	101.63 ± 13.93	<0.001	77.11 ± 13.23	103.05 ± 13.65	<0.001
	73	103		76	113		72	103		74	103	
	58–92	86–126		58–118	96–126		58–92	73–126		58–113	73–126	
HC (cm)	92.03 ± 7.37	117.84 ± 10.09	<0.001	99.62 ± 13.15	124.11 ± 9.99	<0.001	92.76 ± 7.71	115.15 ± 13.32	<0.001	97.63 ± 14.13	113.43 ± 12.7	<0.001
	92	120		97	124		93	114		94	114	
	72–104	100–140		72–128	113–140		72–104	84–140		72–140	84–137	
WHR	0.79 ± 0.05	0.88 ± 0.06	<0.001	0.82 ± 0.06	0.9 ± 0.07	0.002	0.79 ± 0.05	0.88 ± 0.06	<0.001	0.79 ± 0.04	0.9 ± 0.05	<0.001
	0.79	0.87		0.82	0.9		0.78	0.87		0.8	0.88	
	0.7–0.9	0.8–1		0.7–1	0.81–1		0.7–0.9	0.8–1		0.7–0.8	0.9–1	
BODY COMPOSITION												
FFM (kg)	44.08 ± 3.06	53.01 ± 5.35	<0.001	46.99 ± 5.7	53.65 ± 5.49	0.002	44.25 ± 3.11	52.16 ± 5.97	<0.001	45.91 ± 5.49	51.65 ± 5.55	<0.001
	44.14	52.03		46.03	56.1		48.88	51.3		45.54	50.4	
	37.4–49.1	43.5–64.1		37.4–64.1	43.5–62		37.4–49.1	40.4–64.1		37.4–62.6	42.5–64.1	
FFM (%)	74.1 ± 5.63	55.94 ± 4.84	<0.001	68.75 ± 9.07	51.6 ± 3.09	<0.001	74.01 ± 5.98	57.38 ± 6.83	<0.001	71.16 ± 8.97	57.38 ± 6.53	<0.001
	73.71	55.35		70.79	50.95		73.75	55.91		72.9	55.35	
	62.5–84.2	48.1–66.3		53.7–84.2	48.1–57.9		62.5–84.2	48.1–73.6		48.8–84.2	48.1–71.6	
FM (kg)	15.94 ± 5.39	42.81 ± 10.96	<0.001	23.52 ± 12.53	50.99 ± 10.13	<0.001	16.15 ± 5.75	40.59 ± 13.1	<0.001	20.64 ± 12.57	40.08 ± 13.06	<0.001
	15.25	42.74		19.86	54.45		15.03	41.97		15.86	40.21	
	7.5–28.2	23.6–64.4		7.5–54.9	36.6–64.4		7.5–28.2	15.7–64.4		7.5–59.9	17.9–64.4	
FM (%)	25.9 ± 5.63	44.22 ± 5.03	<0.001	31.25 ± 9.07	48.84 ± 3.3	<0.001	25.99 ± 5.98	42.76 ± 6.99	<0.001	28.84 ± 8.97	42.81 ± 6.75	<0.001
	26.29	44.65		29.21	49.46		26.28	44.09		27.1	44.65	
	15.8–37.5	33.7–52		15.8–46.3	42.1–52		15.8–37.5	26.4–52		15.8–51.2	28.4–52	
MM (kg)	19.13 ± 1.51	23.46 ± 2.42	<0.001	20.54 ± 2.73	23.78 ± 2.45	0.002	19.19 ± 1.56	23.08 ± 2.7	<0.001	20.06 ± 2.62	22.74 ± 2.65	<0.001
	19.41	22.9		20.18	24.91		19.41	22.79		19.98	22.5	
	14.8–21.4	19.3–28.3		14.18–28.3	19.3–27.6		14.8–21.4	17.8–28.3		14.8–27.6	18.6–28.3	
VAT (cm^2^)	55.87 ± 29.58	276.4 ± 78.87	<0.001	116.98 ± 97.89	349.33 ± 2	<0.001	53.69 ± 29.98	262.41 ± 90.79	<0.001	86.37 ± 79.78	267.57 ± 100.31	<0.001
	51	296		66	350		51	291		51	301	
	21–116	131–350		21–350	344–350		21–134	88–350		21–350	66–350	
SAT (cm^2^)	89.45 ± 34.97	224.36 ± 75.28	<0.001	118.94 ± 53.71	310.22 ± 43.99	<0.001	91.76 ± 36.05	211.89 ± 84.67	<0.001	105.46 ± 50.07	223.38 ± 88.55	<0.001
	78	202		119	285		78	200		100	219	
	37–194	123–380		37–222	266–380		45–194	37–380		45–267	37–380	
VAT/SAT	0.64 ± 0.36	1.51 ± 0.39	<0.001	0.89 ± 0.5	1.75 ± 0.36	<0.001	0.56 ± 0.13	1.53 ± 0.42	<0.001	0.72 ± 0.33	1.55 ± 0.52	<0.001
	0.55	1.5		0.65	1.89		0.53	1.5		0.57	1.59	
	0.4–2.4	0.9–2.4		0.4–2.4	1.2–2.4		0.4–0.9	0.9–2.4		0.4–1.6	0.5–2.4	
BIOCHEMICAL PARAMETERS												
Fasting glucose (mg/dL)	89.94 ± 5.69	93.74 ± 7.93	NS	92.05 ± 7.38	89.44 ± 3.88	NS	89.66 ± 5.66	93.75 ± 7.72	NS	90.55 ± 5.86	93.43 ± 8.38	NS
	90	93		91.8	90		90	93		90	93	
	77.4–100.8	81–122		77.4–122	81–94		77.4–100.8	81–122		77.4–101	81,122	
Fasting insulin (μU/mL)	5.25 ± 1.95	10.76 ± 4.67	<0.001	7.04 ± 3.84	11.2 ± 5.61	0.007	4.97 ± 1.4	10.66 ± 4.6	<0.001	6.59 ± 3.72	9.58 ± 4.85	0.005
	5	9.4		6.1	10.6		5	9.6		5.8	8.1	
	2.5–11.2	4.8–24.1		2.5–20.1	4.8–24.1		2.5–7.4	4.5–24.1		2.5–20.1	4.5–24.1	
Adiponectin (μg/mL)	10.13 ± 2.77	5.64 ± 2.68	<0.001	8.61 ± 3.57	5.6 ± 1.78	0.013	10.4 ± 2.95	6.68 ± 2.18	<0.001	9.28 ± 3.56	6.2 ± 2.48	<0.001
	10	5.3		8.3	5.9		10.3	5.6		8.8	5.7	
	5.6–16.2	2.2–16.4		3.2–16.4	2.2–7.9		5.6–16.4	2.2–13.2		2.2–16.4	3.2–13.2	
Leptin (ng/mL)	7.97 ± 6.62	22.41 ± 9.52	<0.001	12.7 ± 10.67	23.37 ± 5.9	0.001	7.8 ± 6.81	21.52 ± 9.69	<0.001	8.53 ± 5.83	24.22 ± 9.97	<0.001
	6.3	20.6		9.2	22.5		6.1	20.4		7.7	22.5	
	1.4–32.4	8.3–48.2		1.4–48.2	13.4–33.2		1.4–32.4	8.3–48.2		1.4–21.5	7.8–48.2	
Resistin (ng/mL)	6.16 ± 1.67	8.7 ± 2.66	<0.001	7.01 ± 2.45	8.77 ± 2.37	0.027	6.10 ± 1.71	8.57 ± 2.61	<0.001	6.8 ± 2.68	8.1 ± 1.97	0.002
	5.7	8.1		6.2	8.3		5.7	8		5.9	7.9	
	4.1–13.2	5.3–15.7		4.1–15.7	5.7–11.8		4.1–13.2	5.3–15.7		4.1–15.7	5.5–11.9	
HOMA-IR	1.17 ± 0.46	2.52 ± 1.17	<0.001	1.63 ± 0.98	2.5 ± 1.33	0.014	1.10 ± 0.32	2.49 ± 1.15	<0.001	1.49 ± 0.89	2.24 ± 1.22	0.007
	1.1	2.3		1.3	2.4		1.1	2.3		1.3	1.9	
	0.5–2.5	1.1–5.6		0.5–4.8	1.1–5.6		0.5–1.7	1–5.6		0.5–4.8	1–5.6	
HOMA-AD	2.84 ± 1.57	11.5 ± 5.94	<0.001	5.87 ± 5.68	11.07 ± 5.79	0.005	2.54 ± 0.98	11.18 ± 5.84	<0.001	5.02 ± 5.45	9.51 ± 5.84	<0.001
	2.3	11.4		3.4	11.6		2.3	10.9		2.8	8.3	
	1.2–8.3	2.2–24		1.2–24	3.4–20		1.2–4.6	1.7–24		1.2–24	1.7–23.2	
L/A	0.93 ± 1.13	4.68 ± 2.74	<0.001	2.21 ± 2.68	4.69 ± 2.12	0.002	0.9 ± 1.16	4.44 ± 2.77	<0.001	1.37 ± 1.84	4.66 ± 2.78	<0.001
	0.7	4.3		0.9	4.3		0.6	4.1		0.8	4.6	
	0.1–5.8	0.6–12.1		0.1–12.1	2–9.3		0.1–5.8	0.7–12.1		0.1–9.3	0.7–12.1	
DIET SCORE AND PHYSICAL ACTIVITY												
Diet score	8.39 ± 2.54	6.54 ± 1.95	0.002	7.8 ± 2.52	6.33 ± 1.71	NS	8.21 ± 2.58	6.87 ± 2.16	0.023	8.13 ± 1.19	6.62 ± 2.64	0.026
	9	6		7.5	6		8.5	6.5		8	6	
	1.5–13	4–12		1.5–13	4.5–9		1.5–13	4–12		4–13	1.5–12	
Moderate [min/week]	295.59 ± 143.76	296.64 ± 133.32	NS	301.84 ± 144.1	265.86 ± 101.03	NS	307.57 ± 145.05	283.7 ± 131.49	NS	292.47 ± 136.01	302.04 ± 144.3	NS
	256.8	217.8		256.8	291		256.8	271.8		256.8	293	
	73.7–222.2	71.5–602.7		73.7–611	71.5–379.7		102.2–611	71.5–602.7		73.7–611	71.5–602.7	
Vigorous [min/week]	30.35 ± 46.18	7.71 ± 7.75	0.005	22.98 ± 39.05	5.96 ± 4.67	NS	31.3 ± 47.67	8.37 ± 7.66	0.026	27.8 ± 44.16	7.64 ± 6.62	0.027
	11.2	5.7		9	5.7		11.2	8		10.7	6.3	
	0.2–223.7	0–38.2		0.2–223.7	0–13.2		0.2–223.7	0–38.2		0.2–223.7	0–29.2	
MVPA [min/week]	325.94 ± 146.42	304.36 ± 136.98	NS	234.82 ± 146.78	271.83 ± 104.69	NS	338.87 ± 145.6	292.07 ± 135.27	NS	320.27 ± 139.1	309.69 ± 148.42	NS
	304	273.5		302.3	269.7		239.5	273.5		302.3	301.5	
	82.2–627.3	72.2–609		82.2–627.3	72.2–385.3		106–627.3	72.2–609		82.2–627.3	72.2–609	

Data are mean ± standard deviation, median and interquartile ranges. * Mann–Whitney test; *p* < 0.05. Abbreviations: VAT—visceral fat surface; SAT—subcutaneous fat surface; VAT/SAT—visceral to subcutaneous fat ratio; WHR—waist to hip ratio; BMI—body mass index; WC—waist circumference; HC—hip circumference; FFM—fat-free mass; FM—fat mass; MM—muscle mass; HOMA-IR—homeostatic model assessment of insulin resistance; HOMA-AD—homeostatic model assessment—adiponectin; L/A—leptin to adiponectin ratio; MVPA—moderate to vigorous physical activity; NS—not statistically significant.

**Table 2:** Differences in the prevalence of insulin resistance measured by HOMA-IR, HOMA-AD and L/A divided according to VAT, SAT, VAT/SAT ratio and WHR.

	VAT ≤120 cm^2^ n = 31	VAT >120 cm^2^ n = 25	*p*-Value *	SAT ≤225 cm^2^ n = 47	SAT >225 cm^2^ n = 9	*p*-Value *	VAT/SAT ≤0.9 n = 9	VAT/SAT >0.9 n = 29	*p*-Value*	WHR <0.85 n = 35	WHR ≥0.85 n = 21	*p*-Value *
HOMA-IR ≥ 2.5	1.79%	17.86%	0.002	12.50%	7.14%	NS	0.0%	19.64%	<0.001	5.36%	14.29%	0.013
HOMA-AD ≥ 6.26	3.57%	35.71%	<0.001	26.79%	12.50%	0.021	0.0%	39.29%	<0.001	16.07%	23.21%	0.013
L/A > 2.2	5.36%	39.29%	<0.001	30.36%	14.29%	0.007	5.36%	39.29%	<0.001	12.50%	32.14%	<0.001

* The differences were analyzed with Fisher’s exact test; *p* < 0.05. Abbreviations: VAT—visceral fat surface; SAT—subcutaneous fat surface; VAT/SAT—visceral to subcutaneous fat ratio; WHR—waist-to-hip ratio; HOMA-IR—homeostatic model assessment of insulin resistance; HOMA-AD—homeostatic model assessment—adiponectin; L/A—leptin to adiponectin ratio; NS—not statistically significant.

**Table 3:** Predictors of insulin resistance in PCOS women based on VAT, SAT, VAT/SATand WHR.

Parameters	HOMA-IR	HOMA-AD	L/A
VAT			
OR	20.00	58.00	68.44
95% Cl	2.33–171.18	10.22–329.11	12.57–372.76
*p*-value *	0.002	<0.001	<0.001
SAT			
OR	4.57	7.47	14.11
95% Cl	0.98–21.34	1.38–40.34	1.62–122.70
*p*-value *	NS	0.021	0.007
VAT/SAT			
OR	41.12	241.36	38.13
95% Cl	2.27–743.52	12.67–459.97	8.17–177.85
*p*-value *	<0.001	<0.001	<0.001
WHR			
OR	6.56	4.69	24.00
95% Cl	1.50–28.70	1.47–15.00	5.48–105.05
*p*-value *	0.013	0.011	<0.001

* The odds ratios were analyzed with Fisher’s exact test; *p* < 0.05. Abbreviations: VAT—visceral fat surface; SAT—subcutaneous fat surface; VAT/SAT—visceral to subcutaneous fat ratio; WHR—waist-to-hip ratio; HOMA-IR—homeostatic model assessment of insulin resistance; HOMA-AD—homeostatic model assessment—adiponectin; L/A—leptin to adiponectin ratio; NS—not statistically significant.

## Data Availability

The data analyzed during the current study are available from the corresponding author on reasonable request.
